# Multiplex quantification of four DNA targets in one reaction with Bio-Rad droplet digital PCR system for GMO detection

**DOI:** 10.1038/srep35451

**Published:** 2016-10-14

**Authors:** David Dobnik, Dejan Štebih, Andrej Blejec, Dany Morisset, Jana Žel

**Affiliations:** 1 Department of Biotechnology and Systems Biology, National Institute of Biology, Večna pot 111, 1000 Ljubljana, Slovenia

## Abstract

The advantages of the digital PCR technology are already well documented until now. One way to achieve better cost efficiency of the technique is to use it in a multiplexing strategy. Droplet digital PCR platforms, which include two fluorescence filters, support at least duplex reactions and with some developments and optimization higher multiplexing is possible. The present study not only shows a development of multiplex assays in droplet digital PCR, but also presents a first thorough evaluation of several parameters in such multiplex digital PCR. Two 4-plex assays were developed for quantification of 8 different DNA targets (7 genetically modified maize events and maize endogene). Per assay, two of the targets were labelled with one fluorophore and two with another. As current analysis software does not support analysis of more than duplex, a new R- and Shiny-based web application analysis tool (http://bit.ly/ddPCRmulti) was developed that automates the analysis of 4-plex results. In conclusion, the two developed multiplex assays are suitable for quantification of GMO maize events and the same approach can be used in any other field with a need for accurate and reliable quantification of multiple DNA targets.

After the first report of polymerase chain reaction (PCR) with thermostable DNA polymerase almost three decades ago^1^, it is still widely used in many of its formats. One of such related technologies, quantitative real-time PCR (qPCR), is the most accepted derivative to detect the presence and measure the quantity of the nucleic acid targets. The advents of modern analytical technologies provided the field of nucleic acid quantification with unprecedented sensitivity and specificity, coupled with high accuracy and reproducibility. One of the most exciting developments after real-time PCR is the digital PCR (dPCR) with possibility of measuring the absolute number of targets present in the samples, for which the idea comes already from two decades ago^2^,^3^. Since its introduction, dPCR is getting more and more recognition and the number of publications regarding dPCR has been growing exponentially (see Supplementary Fig. S1). Three different dPCR approaches are available: microfluidic/chip-based dPCR, droplet digital PCR (ddPCR) and combination of both. For all approaches the same principle applies: reaction mixture is divided into hundreds to millions partitions (chambers on chips or droplets in oil emulsion), where each partition undergoes a PCR reaction. At end-point reactions the partitions are scored as positive or negative and these values are used to calculate the target concentration using binomial Poisson statistics^4^.

The use of molecular detection methods is increasing rapidly in various fields of application, such as food control, environmental monitoring, medicine, pharmacy, etc. With growing number of tests performed in the laboratories and in the view of cost efficiency, an important aspect of new technologies is their ability for multiplexing. Possible obstacles with multiplexing are the potential interferences between oligonucleotides and amplification products. Relatively high multiplexing levels are possible with end-point PCR, but the detection methods in such cases might not be as straight forward as gel electrophoresis^5^,^6^,^7^,^8^. Multiplexing in qPCR is limited with the number of filters in the instrument reliably detect fluorescence of different fluorophores, thus the highest level of multiplexing available for now is 5-plex. In dPCR, multiplexing approaches are different between platforms. Some enable detection of three different fluorophores (and 3 targets; Constellation and Naica System) and another five different fluorophores (and 5 targets; BioMark HD). Two other platforms (RainDrop, QX100/200 ddPCR system) enable the detection of two fluorophores to perform duplex reactions, with possibility to perform higher multiplexing (up to 10-plex) in specific reaction setups^9^. Interestingly, one of the reports from 2011 (even before RainDrop platform was officially available) already shows that 5-plex is possible using only two fluorescence channels, based on implementation of different probe concentrations and ratios between both fluorescence labels^10^. Nevertheless, because of just recent explosion of dPCR technology, rare examples of multiplexing (other than duplex) in dPCR have been reported. For the ddPCR platforms the principle of multiplexing can be quite different regarding very different number of droplets per sample or different number of filters. The RainDrop platform, with millions of droplets, works on principle of limiting dilutions, where sample is distributed in partitions in a way that each droplet contains at maximum only one target molecule, whereas for Bio-Rad QX100/200, with around twenty thousand droplets, it is possible for a droplet to contain more target molecules. The possibility of more targets per droplet hampers the analysis as several clusters might appear in analysis panel and their separation might not be as clear as expected. This is probably the reason why for Bio-Rad’s platform the highest multiplexing level reported for now was 3-plex (using hydrolyzing probes^11^ or intercalating dye^12^). The 3-plex with intercalating dye employed separation of droplet clusters based on amplicon length^12^, whereas for hydrolyzing probes the discrimination between targets was based on differentially labelled probes (for two targets) and the use of both fluorescent labels in 50:50 ratio for third target^11^.

In this manuscript the multiplexing with the Bio-Rad’s ddPCR platform was taken one level higher, as we have developed a 4-plex system based on the principle of varying the primers and probes concentration for two targets per fluorescence channel. The Bio-Rad QX ddPCR platform was not initially meant for such high multiplexing and the software, provided with the instrument, did not support the analysis of such experiments. Therefore, a novel analysis tool was developed for fast and automated analysis of raw data. The two multiplex assays reported in this manuscript were developed for the purpose of quantification of genetically modified (GM) maize events. One multiplex assay included targets for one maize endogene and three GM maize events and another for four GM maize events. To follow the European Union regulatory requirements^13^, strict method performance parameters must be considered for a method to be used for detection of genetically modified organisms (GMOs)^14^. Both newly developed 4-plex assays were tested according to these requirements. Overall results have shown good accordance with minimum performance parameters^14^.

## Results and Discussion

### Optimization of primer and probes concentration for duplex reactions in one fluorescence channel

The QX100 Droplet reader used in the studies enables detection of the fluorescence in two different channels (FAM and VIC/HEX)^4^, therefore the duplex amplification is readily available and was shown to perform well^4^,^15^. It was already shown that varying the concentrations of primers has an effect on fluorescence amplitude measured in droplets using DNA-binding dye chemistry^12^. In case of DNA-binding dye chemistry the amplicon length can be used to separate clusters based on fluorescence amplitude^12^,^16^, but in the case of TaqMan hydrolysis probes this was not an option, as the signal corresponds to accumulation of targets and not to the length of amplicons. It was also already reported that the third target can be added to ddPCR with half probes labelled with one fluorophore and another half with second fluorophore^11^. In our study we have addressed yet another option for multiplexing. To increase the multiplexing capabilities of the method, we have decided to exploit the ability of detecting different levels of fluorescence amplitude in droplet readout. Firstly, the goal was to distinguish between the droplets positive for two targets, where probes were labelled with the same fluorescent reporter. In order to reach a clear cluster separation based on fluorescence amplitude, the primers and probes concentrations needed to be optimized. Experiments included different combinations of concentrations of primers and probes for different targets within one fluorescence channel. We aimed at optimal separation of clusters in one fluorescence channel and based on the results, we have selected the most optimal final concentrations (see Supplementary Tables S1 and S2). These concentrations enabled good separation of positive droplets between targets, for which probes were labelled with the same fluorescent reporter and were further used in experiments with multiplex assays. In the performed duplex reactions, a third cluster of positive droplets appeared above the two clusters, which represented droplets positive for each of the targets (see Supplementary Fig. S2). The reason for this was the presence of both targets in individual droplet at the same time, thus the fluorescence increased accordingly. Quantification with these duplex assays (both targets labelled with same fluorophore) was comparable to quantification with simplex assays.

### Duplex to tetraplex with two fluorescent labels

Based on the successful results of duplex experiments, where the droplets containing two amplicons labelled with the same fluorescent reporter could be distinguished and targets quantified, led to the combination of both duplex systems together into one tetraplex reaction for quantification of 4 different targets. The FAM and HEX labelled duplex assays were combined into the tetraplex assays. To show that the tetraplex assays can be combined for multiplex quantification of different targets, two assays were developed, named MTQ1 (targeting GM maize events MON810, DP98140, MON863 and hmgA maize endogene) and MTQ2 (targeting GM maize events MIR162, MIR604, MON89034 and GA21). We have then performed an experiment to test whether the quantification with simplex and tetraplex assays produce comparable results. The DNA sample (DNA mixture of seven above mentioned GM maize events) containing all eight of the amplicons targeted with both assays were used for the comparison. The absolute bias of copies determined with tetraplex assay did not exceed 20%. Although no published criteria or guidelines on comparison of simplex and multiplex methods exist, our results complied with a threshold of 25% that is used for trueness and precision^14^ (Table 1).

### Data analysis tool

The problematic part of such multiplex ddPCR assays is the data analysis, as with four different targets to be quantified in one reaction, there are fifteen different combinations of potential presence of individual target in each droplet, meaning that sixteen clusters of droplets (fifteen positive and one with negative droplets) may appear after droplet readout. Graphic representation of different combinations is presented in Supplementary Fig. S3. Current version of QuantaSoft software does not enable the analysis of more than four clusters of droplets at once, therefore the analysis of the results can be cumbersome and time consuming as two individual exports need to be done and afterward additional calculations must be performed in another software (e.g. Excel spreadsheets). Thus we have developed an interactive web R^17^ and Shiny^18^ based data analysis tool for more user-friendly analysis of tetraplex ddPCR assays (see Supplementary information for ddPCR Calculator manual). The method depends on rather clear separation of sixteen clusters. It calculates positions of cluster separation lines, three in horizontal and three in vertical direction. In cases of poor separation, or less than full number of clusters, user can interactively correct separating line positions to get optimal separation of clusters. Interactive manipulation is aided with visual inspection of reactive cluster display and automatic adaptation of calculated target concentration. Final calculations can be exported as tab delimited text file for further analysis. The tool is available at http://bit.ly/ddPCRmulti. New Bio-Rad’s software, Quantasoft Analysis Pro, which came out during the revision of the manuscript, does support multiplexing, however cluster selection still needs to be performed manually.

### Limit of quantification

The absolute limit of quantification (aLOQ) is the lowest target copy number in a sample that can be reliably quantified with an acceptable level of precision and accuracy^19^. The aLOQ of each amplicon in the ddPCR system was estimated as the lowest copy number within the dynamic range with a relative standard deviation (RSD) of the measured copy number ≤25%^14^. First, preliminary experiments with five replicates, two from the first day and three from the second day, were performed to roughly estimate the aLOQ for each amplicon in both multiplexes. Sample DNA that was used for determination of LOQ was containing all of the targets from both assays. In MTQ1 the preliminary aLOQ was determined to be at least 15 copies for hmgA, 18 copies for DP98140, 44 copies for MON863 and 23 copies for MON810. In MTQ2 the aLOQ was determined to be at least 11 copies for GA21, 49 copies for MIR162, 13 copies for MIR604 and 13 copies for MON89034 (Supplementary Table S3). As these values were compliant with minimum performance parameters^14^ we have performed another experiment with fifteen individual replicates on the selected dilutions around LOQ. The determined aLOQ was to be at least 29 copies for hmgA, 23 copies for DP98140, 36 copies for MON863 and 26 copies for MON810. For MON810, the cv% at LOQ was slightly higher than accepted (28%), however removing one outlying replicate resulted in cv% of 24%, therefore it was considered as accepted. In MTQ2 the aLOQ was determined to be at least 31 copies for GA21, 41 copies for MIR162, 36 copies for MIR604 and 42 copies for MON89034 (Table 2). These results were also comparable to published qPCR results, where some of the aLOQ values were even outside these parameters and ranged from 30 to 100 copies per reaction^20^,^21^.

### Dynamic range

**Figure 1:**
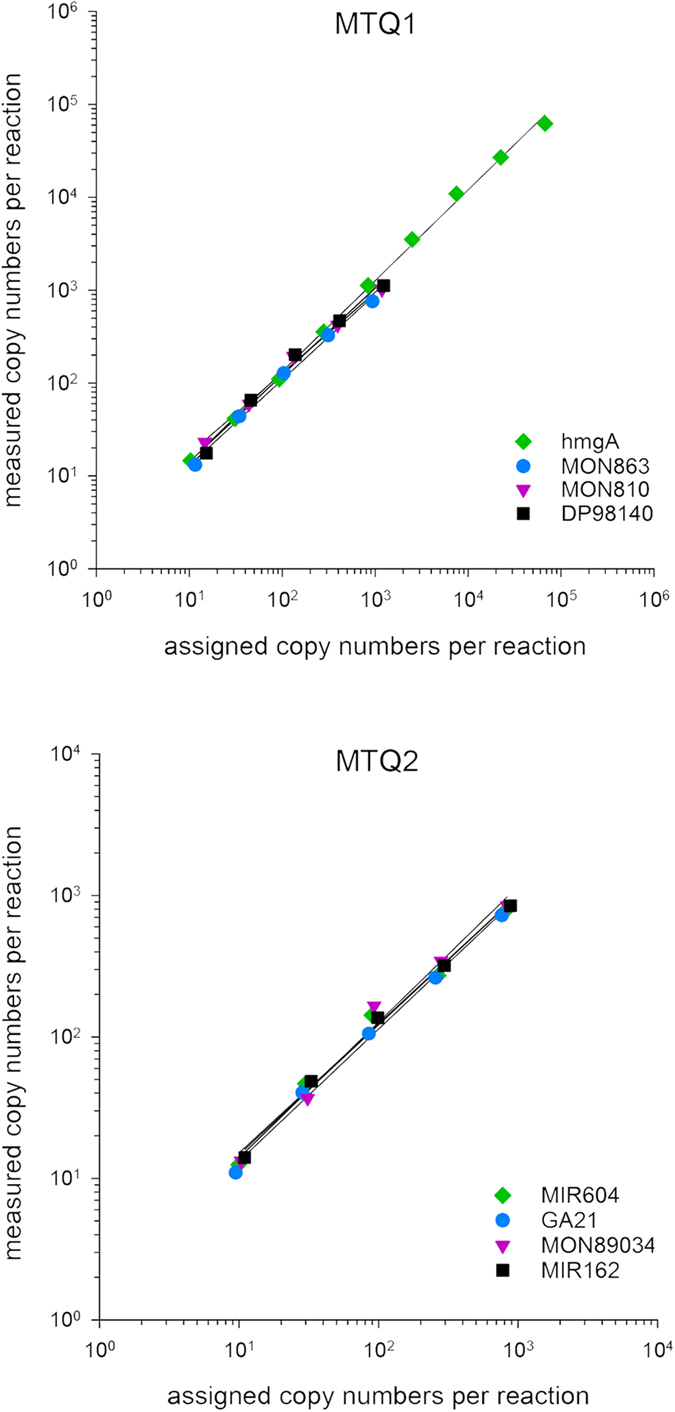
Dynamic range and correlation between measured copy numbers per reaction with the two multiplex assays and assigned copy numbers per reaction on a mixture of 7 maize events. Each data point represents the average of 2 independent experiments with total of 5 replicates. Linear response in the quantitative range was observed for all the tested targets (R^2^ for hmgA, MON863, MON810, DP98140, MIR604, GA21, MON89034 and MIR162 were 0.9885, 0.9920, 0.9922, 0.9913, 0.9954, 0.9988, 0.9901 and 0.9986, respectively).

One of the first studies of ddPCR dynamic range reported covering more than 4 orders of magnitude, whereas the theoretical upper limit is approximately 100,000 target copies^4^. The two developed multiplex assays were investigated over target concentrations ranging from approximately 0.1 to 67,575 hmgA copies and from approximately 0.1 to 1240 individual transgene copies per 20 μL of ddPCR reaction. As the experiments were performed on the mixture of individual targets, originating from DNA of reference materials, the tested copy numbers for GM events could not be as high as for the hmgA endogene. The ddPCR response was linear over whole tested concentration range, however, only the linearity for the range until LOD are presented (Fig. 1). Linear response was shown for hmgA for the concentrations ranging from an average of 10 to 67,575 copies (R2 = 0.9894). Similarly, the ddPCR response for transgenes was, on average, linear from 12 to 950 copies (R2 = 0.9912–0.9990). The results of testing the dynamic range and linearity for the quantification range are presented in Fig. 1. This performance was similar to those reported for MON810 ddPCR assay in simplex or duplex assay^15^ and to multiplex assays for quantification of GM maize in multiplex ddPCR^22^.

### Repeatability

Assessment of the repeatability was done with the data, gathered from the three experiments performed for determination of aLOQ and dynamic range. The RSD of the determined copy numbers all along the quantitative dynamic range was below the acceptance threshold for quantitative methods (RSD < 25%; see Supplementary Tables S4 and S5)^14^. Previous studies have shown that the uncertainty in determined target copies varies across the dynamic range, where the uncertainty and thus the variability was higher when target copies were low, both in chip based dPCR^23^ and ddPCR^4^,^15^. Also in the case of both developed multiplex assays this phenomenon was observed, as the RSD was increasing with the decrease in target copies (see Supplementary Tables S4 and S5).

### Comparison of multiplex ddPCR quantification to qPCR

Both of the developed multiplex assays (MTQ1 and MTQ2) can be used simultaneously to quantify seven transgenic events in two reactions of one run. To estimate the quantification bias of multiplex ddPCR compared to simplex qPCR, we have quantified the DNA mixture also with qPCR. Results of qPCR quantification can be reported in two ways, mass fraction or copy number ratio^24^ and the conversion factor between both is for now set in technical guidance document of EU regulation^25^. This conversion factor is a consensus value to make the conversions easier. However, the real conversion factors for each of the specific maize lines is usually a bit different due to different male and female parent DNA content in endosperm, embryo and aleurone, and can be around 0.4 or 0.6, if GM parent was male or female, respectively. A working group on the Unit of measurement within European Network of GMO Laboratories has been recently established and will try to explain the conversion of copy number ratio to mass fraction, since more biased results can be obtained when using current consensus factor. The results from dPCR are in copy number format, but the results from qPCR are in mass fraction due to the use of reference materials with certified mass fraction. Therefore, for the purpose of direct comparison, the obtained qPCR results were transformed from mass fraction to copy number ratio (see Supplementary Table S6). We have used maize as the testing material and in this case zygosity, maturity status and endosperm tissue ploidy are known biological factors with significant influence on the expressed GMO content in copy number ratio compared to mass/mass ratio estimate using qPCR^26^. This was confirmed also in our experiments, where the bias of multiplex ddPCR compared to values calculated with the use of technical guidance document, in some cases deviated for more than 25% (see Supplementary Table S6). Whereas when taking into account the parental origin of the material^26^ and the experimentally determined factors, the bias of the multiplex system is more uniform and deviated less than 25% (see Supplementary Table S6). By this comparison we have shown that the quantification with digital PCR multiplex system is completely comparable to simplex qPCR quantification.

### Sensitivity

The sensitivity was assessed by determining the absolute limit of detection (aLOD), which is the lowest target copy number in a sample that can be reliably detected, but not necessarily quantified^14^. The data from repeatability experiment with fifteen replicates were used to determine the lowest concentration level for which at least fourteen ddPCR replicates resulted in a positive reaction (at least three positive droplets per target). In MTQ1 the aLOD was determined to be at 10 copies for hmgA, 17 copies for DP98140, 12 copies for MON863 and 18 copies for MON810. In MTQ2 the aLOD was determined to be at 9 copies for GA21, 11 copies for MIR162, 10 copies for MIR604 and 11 copies for MON89034. These results are compliant with the performance requirements for GMO testing methods in the EU (i.e. aLOD < 25 copies)^14^. The absolute sensitivity for MON810 is comparable to the one observed for simplex qPCR assay at around 6–18 copies and in microfluidic/chip-based digital PCR (BiomarkHD, Fluidigm)^27^. In view of the EU Regulation 619/2011^28^ it is important to evaluate possible influence of high concentrations of some targets in the sample on the amplification of targets at low concentrations (e.g. GM events pending for authorization or the ones for which the authorization has expired, for which a threshold in EU is set at 0.1%). Therefore, additional experiments, using asymmetric target concentrations in the samples, were performed. We have reliably detected all of the individual targets present at low concentration (at 20 and 40 copies per reaction) in the mix of other targets at higher concentrations (at least 70,000 copies of which 56,000 were hmgA and others the GM targets). These results suggest that highly concentrated targets are not interfering with amplification of targets at low concentration as we could detect targets at concentrations lower than 0.1% in high background of others. This reduction of interactions between targets is possible in ddPCR due to the separation of reaction mixture into thousands of partitions, where after distribution into individual partitions majority of targets are separated and are present in lower amount in individual partitions.

### Specificity

The specificity of both multiplex ddPCR assays was assessed on different mixtures of target DNAs, where one or more targets were not present. The acceptance values of ≤5% false negative rate are widely adopted^29^ and was translated also to a false positive rate ≤5%. In present experiments no false-negative results were observed over all replicates of positive samples. At specific conditions we have observed false positive results for the target with positive droplet cluster with lower fluorescence amplitude. This happened due to the rain effect (intermediate fluorescence of some droplets), which can be observed with ddPCR, as its presence is correlated with target DNA concentration^30^,^31^. These ‘‘rain’’ droplets originating from targets with high fluorescence amplitude droplet cluster can fall inside the space of supposed droplet cluster for target with low fluorescence amplitude, thus giving a false positive signal for the low fluorescence target (example in Supplementary Fig. S4). Possible false positive signal, however, does not contribute significantly to the final quantification, whenever both of the targets (targeted with probes labelled with same fluorophore) are present in the sample. To determine the presence of false positives, individual DNA templates were used and run with multiplex primer and probe sets. In all cases it was observed that the number of these false positive droplets is usually <1% of the total positive droplets for target producing high fluorescence amplitude droplet cluster. For the purpose of GMO quantification this does not pose a problem, since a high concentration of one target exceeding 0.9% threshold would automatically mean that the product must be labelled, independently of the presence of other GM events at lower concentrations (be it true positive or false positive). At and below concentration of 300 copies per reaction the false positive droplets disappear. It should be kept in mind that false positive droplets can occur and results need to be handled appropriately. Nevertheless, the multiplex assays are in a limited range still completely suitable for the purpose of GMO quantification.

### Fitness for purpose

The multiplex assays were developed for a purpose of quantification of seven GM maize events after positive screening results. The two developed assays were used to assess the closeness of agreement between real GM% value (certified or determined with qPCR) and multiplex ddPCR result on different samples: certified reference materials, proficiency test samples and real-life samples from official control. The same samples were previously tested with qPCR within official testing procedure under accredited quality assurance system. Running MTQ1 or MTQ2 on the reference materials in all cases, after applying the conversion factor discussed above, resulted in the GM content comparable to the certified value (Supplementary Table S7). Z-score is the measure used in evaluating the results of individual laboratories in proficiency tests. The assigned Z-score indicates the statistical distance of results from the mean GM% value calculated from results of all of the participating laboratories (robust mean). All values where |Z| < 2 pass the proficiency test. In the three proficiency test samples used in this study a total of 10 individual quantifications were made. In all of the ten cases |Z| < 2 (see Supplementary Table S8), therefore the results of the multiplex ddPCR quantification would have passed the test. It can be noted that this threshold is a bit less strict than the 25% bias mentioned above. Although the absolute bias of multiplex ddPCR to robust mean in some cases exceeds 25%, this can be noticed also in the case of qPCR, where the results for six of quantifications were also compliant with the |Z| < 2 threshold. Additionally, a comparison of quantification with multiplex ddPCR and qPCR was made on two real life samples, containing two GM maize events (see Supplementary Table S9). In these cases, the bias between ddPCR and qPCR was within 25% when actual conversion factors were used for conversion of mass/mass qPCR results to copy numbers. However, due to high concentration of MON810 event in the samples and the rain of droplets, we observed a false positive result for DP98140, which was still below the 0.9% threshold for labelling. As discussed within Specificity paragraph, the amount of false positives would not have an effect on the compliance with regulation on labelling, thus this kind of result could still be acceptable. But this can be a problem for events pending for authorization or for which the authorization has expired, for which a lower threshold of 0.1% is established in the EU. At the time of development DP98140 was still approved in EU, but is now not approved anymore. Therefore, in case of positive DP98140 result, it would anyway need to be confirmed and quantified with qPCR, since the regulation requires a mass/mass GM percentage, which can (without implementing any conversion) be achieved with qPCR. Nevertheless, the basic idea behind the developed multiplex assays allows preparation of new assays, which could cover only the approved events and our results suggest that the performance of these new assays would be similar to the ones reported.

### Summary

None of the published studies of ddPCR multiplexing has yet reported a thorough characterization taking into account several performance parameters, like limits of quantification and detection, sensitivity, repeatability and specificity. Therefore, the present study serves as a pioneer example of true multiplexing capabilities of ddPCR. The parameters of quantification with the two newly developed 4-plex assays were tested against strict minimum performance parameters^14^ used in the field of GMO diagnostics in EU. All of the tested parameters were compliant with the performance requirements for GMO testing methods in the EU^14^ with some exceptions for specificity. When all targets are present at relatively high concentrations, as was usually the case in already reported studies, the new multiplex assays perform well, but as shown here, there are still some problems in terms of false positives, when some targets are missing and other are present in high concentration, due to the rain effect. Thus, there is still some space for future improvements of ddPCR reactions, most probably in terms of improving the chemistry of reaction, to increase the resolution of cluster separation, cluster bandwidth and eliminating the so-called rain effect. Additionally, we have shown that with the development of new data analysis tool, previously cumbersome analysis with manual selection of clusters and several exports was now made simple and straightforward. Besides the absolute concentration of the targets, part of the output of the new tool is also the necessary statistical analysis. Nevertheless, the present report shows the potential of multiplexing in ddPCR in terms of simultaneous repeatable and reliable absolute quantification of (at least) four targets.

## Methods

### Test material

A list of all certified reference materials (CRMs) used in this study is provided in Supplementary Table S10. They all have certified mass/mass (m/m) GM maize/wild-type maize material ratios. DNA extraction was performed as described previously^22^. A specificity study was conducted on DNA mixtures of reference materials containing 2, 3, 4 or seven targets from the multiplex assays. Fitness for purpose was assessed using individual CRMs, real life samples characterized by qPCR and with proficiency test samples from USDA/GIPSA Proficiency Program of 2008 and 2009, all performed in two replicates.

### Primers and probes mix preparation

The hmgA gene was used as the endogenous reference gene for maize, to which relative quantity of the GM events was estimated. The existing simplex qPCR EURL validated methods (see Supplementary Table S11) served as a base for development of ddPCR multiplex assays. Nucleotide sequences of primers and probes used are presented in Supplementary Tables S1 and S2. Primers and probe concentrations as in methods referenced in Supplementary Table S11 were used in simplex ddPCR to determine the GM content in each sample. Testing of duplex ddPCR assays in one fluorescence channel included different combinations of primer and probe concentrations of original simplex methods. Combinations of concentrations 300 and 900 μM for primers and 100, 200 (only for DP98140) and 300 μM for probes were used. The preparation of multiplex ddPCR target quantification assay 1 (MTQ1) and 2 (MTQ2) primer and probe (PP) mixes was done as follows. For MTQ1, primers and probes for the endogene (hmgA) and three events - MON810, MON863, DP98140 were mixed (for final concentrations see Supplementary Table S1). For MTQ2, primers and probes for the events GA21, MIR162, MIR604 and MON89034 were mixed (for final concentrations see Supplementary Table S2). Duplex ddPCR reactions in one channel contained the primers and probes concentration as in tetraplex, but only the assays with same fluorescent label were used. Primers and probes were purchased from Eurofins MWG Operon (Ebersberg, Germany) or from Integrated DNA Technologies (Leuven, Belgium). Primers and probes were shipped lyophilized and diluted in nuclease- and protease-free water (Sigma-Aldrich Chemie GmbH, Munich, Germany) upon reception.

### Droplet digital PCR reactions and data analysis

The reaction mix for all reactions, simplex as well as multiplex, was composed of and prepared as described previously^22^, but with different primers and probes mixes (see above for details). Likewise the reaction conditions and data acquisition was as already reported^22^. The analysis was performed using QuantaSoft software (v1.6.6.0320, Bio-Rad, Pleasanton, CA) as follows. Positive droplets, containing amplification products, were discriminated from negative droplets without amplification products by applying a fluorescence amplitude threshold. The threshold was set manually, using the 2D amplitude chart. The threshold was always set in the middle between the clusters (see example on Supplementary Fig. S5). The lasso tool was used to select for corresponding positive clusters and two exports needed to be performed for each reaction to get the data for all four targets (Supplementary Fig. S5). For each analysis, first the positive control (with all of the major clusters present) was analysed to establish the approximate value for thresholds between the clusters. The information on specific thresholds for the assays is not reported, since it would not give any additional information for tests in another lab, because the measured fluorescence amplitude depends on different things, such as batch of probes and droplet reader calibration. For more details on this topic see “ddPRC Calculator manual” in Supplementary information. After being exported as comma separated value (.csv) files, the data were further analysed in Microsoft Excel spreadsheets. The number of template copies per μL was calculated from the values of positive and accepted droplets using the volume of 0.85 nL per droplet. Digital MIQE guidelines^32^ were followed. For the purpose of data analysis with the newly developed automated tool raw fluorescence data for each well were exported.

### Comparison of simplex, duplex and tetraplex quantification

The performance of ddPCR MTQ1 and MTQ2 multiplex assays was compared to simplex assays. Both hmgA simplex and event simplex reactions were performed in a duplicate on individual DNA samples. The MTQ1 and MTQ2 assays were also tested on individual DNAs in duplicates. Duplex assays were tested on a mixture of DNAs including at least the two targeted amplicons.

### Dynamic range, repeatability, limits of detection and quantification

A dilution series in terms of target copies was prepared from mixture of DNAs of all seven transgenic maize events extracted from the CRMs (each event was present approximately in equal number of target copies). The quantity of each GM maize event in the DNA mix solution was determined by simplex ddPCR (individual events and hmgA) on undiluted DNA mix in duplicates. These values were used to calculate the assigned copy number of targets in the dilution series of DNA mix. The specifications of the DNA mixture are presented in Supplementary Table S12. Five replicates of the dilution series were measured by ddPCR for preliminary experiment (two separate runs, two replicates first day and three replicates second day). The determination of absolute limit of quantification (aLOQ, in copy numbers) and absolute limit of detection (aLOD) for ddPCR were determined based on these experimental results of third set of experiments performed on fifteen replicates. The LOQ was determined as the aLOQ in the sample, where the relative standard deviation (RSD) of all replicates was below 25%. The LOD was determined as the aLOD in the sample, where at least fourteen replicates still produced a positive signal. Three droplets were used as a threshold for positive signal. Two of the outliers, determined based on the value above 1.5 times the interquartile range, were discarded (one value for DNA mix 3 for MON863 and one value for DNA mix 4b for DP98140).

### Sensitivity

Dilution series of DNA mixture described above was used to assess sensitivity in terms of aLOD. Additionally, to assess whether low copy number of targets can be detected in background of high concentration of other targets (asymmetric LOD [LODasym]^33^), different DNA mixtures with low concentration of one of the targets were prepared. Two different levels of samples with low target concentration for LODasym were tested, each containing all 8 targets of which one was at approximately 40 (first level) and another at 20 (second level) copies per reaction. This was not possible for hmgA target since it is not possible to get higher concentration of GM event than the endogene. On average, excluding the target at 40 or 20 copies, the mixtures contained the following concentration of target copies per reaction: 56378 of hmgA, 186 of MON863, 136 of MON810, 278 of DP98140, 9735 of MON89034, 1715 of MIR604, 18537 of MIR162 and 1573 of GA21. Moreover, DNA mixtures lacking one of the targets were also prepared and tested.

### Automated data analysis tool

To support analysis of results obtained by QuantaSoft, we developed a semi-automatic interactive analysis tool. This R^17^ and Shiny^18^ based web application enables users to upload their own data, visually inspect the automatically proposed classification of points and, if necessary, interactively fine tune the classification. Final calculations of target concentration with corresponding confidence intervals are based on the confirmed classification and can be downloaded into text files for further analysis. The analysis tool can be accessed at http://bit.ly/ddPCRmulti. For instructions see the ddPCR Calculator manual in the Supplementary information.

## Additional Information

**How to cite this article**: Dobnik, D. *et al*. Multiplex quantification of four DNA targets in one reaction with Bio-Rad droplet digital PCR system for GMO detection. *Sci. Rep.* **6**, 35451; doi: 10.1038/srep35451 (2016).

## Supplementary Material

Supplementary Information

## Figures and Tables

**Table 1:** Performance comparison of tetraplex ddPCR assay against simplex conditions on DNA mixture of 7 GM maize events.

GM event	Average copy number		Bias tetraplex to simplex (%)
	Simplex	Tetraplex	
MON863	936	755	−19.4
MON810	1184	1022	−13.7
DP98140	1237	1119	−9.5
MIR604	803	769	−4.3
GA21	767	721	−6.0
MON89034	837	849	1.5
MIR162	887	848	−4.4
hmgA	67576	62109	−8.1

**Table 2:** Limits of quantification and detection for individual targets (in target copies per reaction) covered by the multiplex assays MTQ1 and MTQ2 calculated from fifteen replicates.

Sample	hmgA	MON863	MON810	DP98140	MIR604	GA21	MON89034	MIR162
DNA mix 3	10079	121	176	175	123	105	134	131
DNA mix 4	3174	36^a^	58	58	36^a^	31^a^	42^a^	41^a^
DNA mix 4a	1536	17	26^a^	29	n.t.	n.t.	n.t.	n.t.
DNA mix 4b	1233	15	21	23^a^	n.t.	n.t.	n.t.	n.t.
DNA mix 5	993	12^b^^,^^c^	18^b^	17^b^	10^b^	9^b^^,^^c^	11^b^	11^b^
DNA mix 6	281	neg	neg	neg	neg	neg	neg	neg
DNA mix 7	96	neg	neg	neg	neg	neg	neg	neg
DNA mix 8	29^a^	neg	neg	neg	neg	neg	neg	neg
DNA mix 9	10^b^^,^^c^	neg	neg	neg	neg	neg	neg	neg

^a^Limit of quantification.

^b^Limit of detection.

^c^One of fifteen replicates was negative with one or two positive droplets; neg – at least two replicates out of fifteen was negative.
