# Supplementary material for: Multiplex quantification of four DNA targets in one reaction with Bio-Rad droplet digital PCR system for GMO detection
Source: Sci Rep. 2016 Oct 14;6:35451. doi: 10.1038/srep35451 (PMC5064307; doi:10.1038/srep35451)
Supplement: Supplementary Information [file srep35451-s1.pdf]

Supplementary information for:

Multiplex quantification of four DNA targets in one reaction with Bio-Rad droplet digital PCR system for GMO detection

David Dobnik*, Dejan Štebih, Andrej Blejec, Dany Morisset, Jana Žel

Department of Biotechnology and Systems Biology, National Institute of Biology, Večna pot 111, 1000 Ljubljana, Slovenia

***Corresponding author:** david.dobnik@nib.si

Table of contents:

Table S1. Primers and probes used in the MTQ1 assay 2

Table S2. Primers and probes used in the MTQ2 assay 3

Table S3. Preliminary limits of quantification and detection for individual targets (in target copies per reaction) covered by the multiplex assays MTQ1 and MTQ2 calculated from five replicates 4

Table S4. Results of preliminary experiments for target copies determination with MTQ1 and MTQ2 assay on a dilution series (evaluating preliminary absolute limits of quantification and detection) 5

Table S5. Results of experiment for evaluating absolute limits of quantification and detection with MTQ1 and MTQ2 assays 6

Table S6. Comparison of quantification result of multiplex ddPCR to qPCR 7

Table S7. Evaluating fitness for purpose with certified reference materials 8

Table S8. Evaluating fitness for purpose with proficiency test samples. 9

Table S9. Testing of both multiplex assays on real life samples in comparison to qPCR 10

Table S10. List of all CRM test materials used in this study 11

Table S11. Methods used in this study 12

Table S12. Specifications of stock DNA mix and all of the dilution series samples 13

Figure S1. Number of publications containing “digital PCR” (blue line) or “droplet digital PCR” (green line) in title, abstract or keywords from 1992 to the end of 2015 gathered from “Analyze search results” module in Scopus database 14

Figure S2. Example of duplex droplet readout in one fluorescence channel 15

Figure S3. Graphical representation of possible target combinations in droplet clusters and their area of appearance in the 2-D amplitude view 16

Figure S4. Example of false positive signal in the case of MTQ2 assay 17

Figure S5. Example of cluster selection with the lasso tool 18

ddPCR Calculator manual 19

Reference list 22

Table S1. Primers and probes used in the MTQ1 assay

Name	DNA sequence of the oligonucleotide (5'-sequence-3')	Final concentration in the PCR [nmol/L]
hmgA as the target sequence:		
Fw-hmgA	TTGGACTAGAAATCTCGTGCTGA	300
R-hmgA	GCTACATAGGGAGCCTTGTCCT	300
P-hmgA	6-FAM-CAATCCACACAAACGCACGCGTA-BHQ-1	100
DP98140 as the target sequence:		
Fw-DP98140	GTGTGTATGTCTCTTTGCTTGGTCTT	300
R-DP98140	GATTGTCGTTTCCCGCCTTC	300
P-DP98140	HEX-CTCTATCGATCCCCCTCTTTGATAGTTTAAACT-BHQ-1	200
MON810 as the target sequence:		
Fw-MON810	TCGAAGGACGAAGGACTCTAACGT	900
R-MON810	GCCACCTTCCTTTTCCACTATCTT	900
P-MON810	HEX-TCTAGACAATTCAGTACATTAAAAACGTCCGCCA-BHQ-1	300
MON863 as the target sequence:		
Fw-MON863	GGGATAAGCAAGTAAAAGCGCTC	900
R-MON863	CCTTAATTCTCCGCTCATGATCAG	900
P-MON863	6-FAM-TTTAAACTGAAGGCGGGAAACGACAA-BHQ-1	300

6-FAM: 6-carboxyfluorescein; BHQ1: Black Hole Quencher 1; HEX: hexachloro-6-carboxyfluorescein.

The sequences of primers and probes are from the EURL validated methods for quantification of event (see Table S-8 for reference details).

Table S2. Primers and probes used in the MTQ2 assay

Name	DNA sequence of the oligonucleotide (5'-sequence-3')	Final concentration in the PCR [nmol/L]
GA21 as the target sequence:		
Fw-GA21	CGTTATGCTATTTGCAACTTTAGAACA	900
R-GA21	GCGATCCTCCTCGCGTT	900
P-GA21	HEX-TTTCTCAACAGCAGGTGGGTCCGGGT-BHQ-1	300
MIR162 as the target sequence:		
Fw-MIR162	GCGCGGTGTCATCTATGTTACTAG	300
R-MIR162	TGCCTTATCTGTTGCCTTCAGA	300
P-MIR162	HEX-TCTAGACAATTCAGTACATTAAAAACGTCCGCCA-BHQ-1	300
MIR604 as the target sequence:		
Fw-MIR604	GCGCACGCAATTCAACAG	300
R-MIR604	GGTCATAACGTGACTCCCTTAATTCT	300
P-MIR604	6-FAM-AGGCGGGAAACGACAATCTGATCATG-BHQ-1	300
MON89034 as the target sequence:		
Fw-MON89034	TTCTCCATATTGACCATCATACTCATT	300
R-MON89034	CGGTATCTATAATACCGTGGTTTTTAAA	300
P-MON89034	6-FAM-ATCCCCGGAAATTATGTT-MGBNFQ	100

6-FAM: 6-carboxyfluorescein; BHQ1: Black Hole Quencher 1; HEX: hexachloro-6-carboxyfluorescein; MGBNFQ: Molecular-Groove Binding Non-fluorescence Quencher.

The sequences of primers and probes are from the EURL validated methods for quantification of event (see table S-8 for reference details).

Table S3. Preliminary limits of quantification and detection for individual targets (in target copies per reaction) covered by the multiplex assays MTQ1 and MTQ2 calculated from five replicates

Sample	hmgA	MON863	MON810	DP98140	MIR604	GA21	MON89034	MIR162
DNA mix 1	62109	755	1022	1119	769	721	849	848
DNA mix 2	26905	324	418	467	272	261	344	319
DNA mix 3	10905	127	194	202	143	106	166	137
DNA mix 4	3525	44 [a]	59	65	47	40	37	49 [a]
DNA mix 5	1128	13 [b]	23 [a,b]	18 [a,b]	12 [a,b]	11 [a,b]	13 [a,b]	14 [b]
DNA mix 6	356	neg	neg	neg	neg	neg	neg	neg
DNA mix 7	110	neg	neg	neg	neg	neg	neg	neg
DNA mix 8	41	neg	neg	neg	neg	neg	neg	neg
DNA mix 9	15 [a,b]	neg	neg	neg	neg	neg	neg	neg

[a] limit of quantification; [b] limit of detection; neg – at least one replicate out of five was negative

Table S4. Results of preliminary experiments for target copies determination with MTQ1 and MTQ2 assay on a dilution series (evaluating preliminary absolute limits of quantification and detection)[a]

MTQ1	MON863						MON810						DP98140						hmgA					
Sample	average day 1	cv% day 1	average day 2	cv% day 2	average	cv %	average day 1	cv% day 1	average day 2	cv% day 2	average	cv %	average day 1	cv% day 1	average day 2	cv% day 2	average	cv %	average day 1	cv% day 1	average day 2	cv% day 2	average	cv %
DNA mix 1	758	2	752	11	755	7.8	1077	3.5	985	13.5	1022	10.6	1157	3.6	1094	15.9	1119	11.5	63269	2.3	61336	10.0	62109	7.3
DNA mix 2	329	9	321	10	324	8.6	383	10.3	442	9.1	418	11.4	474	1.1	463	5.5	467	4.1	25610	1.4	27768	1.7	26905	4.6
DNA mix 3	139	7	119	19	127	15.8	205	10.4	186	13.0	194	11.7	189	41.3	211	3.9	202	20.4	10982	2.2	10854	0.7	10905	1.4
DNA mix 4	39	1	46	14	44	13.6	52	3.7	64	5.5	59	12.0	70	1.1	62	3.9	65	7.1	3476	2.2	3558	2.8	3525	2.6
DNA mix 5	19	12	9	49	13	49.1	23	12.3	23	12.2	23	10.6	20	30.9	16	11.4	18	22.5	1094	6.4	1151	2.8	1128	4.6
DNA mix 6	neg		neg		neg		neg		neg		neg		7	3.9	neg		neg		371	8.8	345	8.6	356	8.4
DNA mix 7	neg		neg		neg		neg		neg		neg		neg		neg		neg		118	5.2	105	22.9	110	17.0
DNA mix 8	neg		neg		neg		neg		neg		neg		neg		neg		neg		40	6.7	42	29.6	41	21.6
DNA mix 9	neg		neg		neg		neg		neg		neg		neg		neg		neg		14	38.9	15	17.6	15	22.9
DNA mix 10	neg		neg		neg		neg		neg		neg		neg		neg		neg		neg		neg		neg	

MTQ2	MIR604						GA21						MON89034						MIR162					
Sample	average day 1	cv% day 1	average day 2	cv% day 2	average	cv %	average day 1	cv% day 1	average day 2	cv% day 2	average	cv %	average day 1	cv% day 1	average day 2	cv% day 2	average	cv %	average day 1	cv% day 1	average day 2	cv% day 2	average	cv %
DNA mix 1	734	10.0	792	4.6	769	7.2	734	1.0	712	2.7	721	2.6	855	2.3	846	5.0	849	3.8	812	3	872	6	848	6
DNA mix 2	254	5.8	284	1.0	272	6.7	260	4.1	262	7.9	261	5.9	353	7.4	339	5.7	344	5.9	300	14	331	5	319	9
DNA mix 3	147	3.1	141	9.5	143	7.2	84	20.4	120	12.2	106	22.5	166	1.9	166	8.6	166	6.1	143	15	133	10	137	11
DNA mix 4	52	7.6	42.9	16.9	47	16.2	41	36.1	40	12.6	40	20.4	39	11.3	35	27.3	37	20.3	54	13	45	28	49	22
DNA mix 5	12	19.3	12.9	7.8	12	11.7	9	20.0	12	8.5	11	19.4	12	27.8	14	20.3	13	21.7	15	24	13	51	14	37
DNA mix 6	neg		neg		neg		neg		neg		neg		neg		neg		neg		neg		neg		neg	

[a] showing only the results, where all replicates are positive at individual day or altogether.

Individual samples represent the same DNA mix, containing all 7 GM maize lines that were tested, in a serial dilution. Values considered as both aLOQ and aLOD are highlighted in green, value considered as aLOQ is highlighted in blue and value considered as aLOD is highlighted in orange.

Table S5. Results of experiment for evaluating absolute limits of quantification and detection with MTQ1 and MTQ2 assays[a]

MTQ1	MON863		MON810		DP98140		hmgA	
Sample	average	cv %	average	cv %	average	cv %	average	cv %
DNA mix 3	121	12	176	10.6	175	8.0	10079	2.1
DNA mix 4	36	19	58	17.2	58	15.3	3174	4.4
DNA mix 4a	17	31	26	28.3[b]	29	24.9	1536	3.3
DNA mix 4b	15	26	22	28.4	23	24.4	1233	3.9
DNA mix 5	12	37	18	22.7	17	25.9	993	4.4
DNA mix 6	neg		neg		neg		281	7.8
DNA mix 7	neg		neg		neg		96	10.7
DNA mix 8	neg		neg		neg		29	21.7
DNA mix 9	neg		neg		neg		10	29.9
DNA mix 10	neg		neg		neg		neg	

MTQ2	MIR604		GA21		MON89034		MIR162	
Sample	average	cv %	average	cv %	average	cv %	average	cv %
DNA mix 3	123	10.9	105	12.3	134	8.1	131	9
DNA mix 4	36	23.6	31	23.7	42	20.7	41	18
DNA mix 5	10	54.3	9	34.4	11	31.1	11	41
DNA mix 6								

[a] showing only the results, where at least fourteen out of fifteen replicates were positive.

[b] by removing one outlier, the cv % is 24.1%, therefore this dilution is considered as LOQ.

Individual samples represent the same DNA mix, containing all 7 GM maize lines that were tested, in a serial dilution. Values considered as aLOQ is highlighted in blue and value considered as aLOD is highlighted in orange.

Table S6. Comparison of quantification result of multiplex ddPCR to qPCR

GM event	GM% ddPCR[b]	GM%[a] qPCR[c]	% bias of ddPCR to qPCR	GM%[a] qPCR[d]	% bias of ddPCR to qPCR	Factor used for conversion[e]
MON810	1.7	2.1	-15.8	1.6	7.99	0.39
MON863	1.2	1.1	8.0	1.4	-12.9	0.62
DP98140	1.8	1.0	79.1	1.6	13.4	0.79
MIR604	1.2	1.3	-9.0	1.0	19.7	0.38
MON89034	1.3	1.0	32.3	1.2	8.4	0.61
GA21	1.0	1.5	-29.0	1.0	4.5	0.34
MIR162	1.3	1.0	25.5	1.2	6.38	0.59

GM% is reported as copy number ratio (copies of transgene/copies of endogene).

[a] values represent an average of measures of 2 replicates from 3 dilutions

[b] values represent an average GM% calculated from average copy numbers of 5 replicates for each of the dilutions within aLOQ range

[c] as qPCR results are usually reported in mass/mass ration they needed to be converted to copy number ratio. Conversion factor of 0.5 from EURL Technical guidance on implementation of EU Regulation 619/2011[1] was used to convert qPCR results from mass/mass ratio to copy number ratio in this column.

[d] as in [c] the qPCR results were converted to copy number ratio, but in this column the conversion factor, determined based on experimental results, was used (certified reference materials were subjected to absolute quantification with ddPCR and then copy number result was compared to the certified mass/mass %)

[e] conversion factors determined based on experimental results (certified reference materials were subjected to absolute quantification with ddPCR and then copy number result was compared to the certified mass/mass %)

Table S7. Evaluating fitness for purpose with certified reference materials

DNA sample	GM % certified value	GM % with ddPCR multiplex	conversion factor[a]	determined GM %	% bias ddPCR to certified
DP98140	2.0	1.64	0.79	2.08	3.80
MON810	9.9	3.72	0.39	9.54	-3.65
MON810[b]	0.77	0.83	1.00	0.83	7.79
MON863	9.85	6.09	0.62	9.82	-0.28
GA21	99.98	27.06	0.34	79.59	-20.40
MIR162	99.88	54.78	0.59	92.85	-7.04
MIR604	99.98	32.51	0.38	85.55	-14.43
MON89034	99.425	57.01	0.61	93.46	-6.00

[a] conversion factors determined based on experimental results (certified reference materials were subjected to absolute quantification with ddPCR and then copy number result was compared to the certified mass/mass %; for factors see Table S6) were used to convert ddPCR results from copy number ratio to mass/mass ratio

[b] the material was certified for copy number ratio (transgene/endogene)

Table S8. Evaluating fitness for purpose with proficiency test samples.

Sample[a]	GM event	Robust mean	GM% qPCR quantification	Bias to robust mean [%]	Z-score for qPCR	GM %ddPCR quantification[b]	Bias to robust mean [%]	Z-score for ddPCR	GM% ddPCR quantification[c]	Bias to robust mean [%]	Z-score for ddPCR
181/08	MON863	0.56	0.50	-10.2	-0.3	0.46	-16.7	-0.5	0.37	-32.8	-0.9
	MON810	0.13	<0.10			0.04	-68.3	-0.6	0.05	-59.3	-0.5
	MIR604	1.10	0.60	-45.2	-1.0	0.93	-14.7	-0.3	1.23	12.2	0.3
	GA21	0.35	0.20	-42.6	-1.0	0.22	-37.0	-0.9	0.32	-7.4	-0.2
188/09	MON810	0.63				0.55	-13.0	-0.3	0.70	11.5	0.3
	MIR604	0.39	0.30	-23.7	-0.9	0.40	0.7	0.0	0.52	32.5	1.2
	GA21	0.19	0.14	-27.4	-0.8	0.21	7.6	0.2	0.31	58.3	1.8
190/09	MON863	0.62				0.70	12.7	0.5	0.56	-9.1	-0.3
	MON810	0.53				0.43	-19.3	-0.5	0.55	3.4	0.1
	GA21	0.29	0.09	-68.9	-1.7	0.34	17.4	0.4	0.50	72.6	1.8

[a] samples of maize flour from USDA/GIPSA proficiency program of 2008 and 2009

[b] conversion factor of 0.5 from EURL Technical guidance on implementation of EU Regulation 619/2011[1] was used to convert ddPCR results from copy number ratio to mass/mass ratio

[c] conversion factors determined based on experimental results (certified reference materials were subjected to absolute quantification with ddPCR and then copy number result was compared to the certified mass/mass %; for factors see Table S6) were used to convert ddPCR results from copy number ratio to mass/mass ratio

Table S9. Testing of both multiplex assays on real life samples in comparison to qPCR

Sample	GM event	GM% qPCR quantification	GM% ddPCR quantification[a]	Bias of ddPCR to qPCR [%]	ddPCR quantification[b]	Bias of ddPCR to qPCR [%]
G187/14	MON810	27.0	20.0	-26.1	25.6	-5.2
	MON89034	54.0	81.3	50.5	66.6	23.4
	DP98140		0.2		0.1	
G189/14	MON810	62.0	57.3	-7.7	73.4	18.4
	MON89034	36.0	42.2	17.2	34.6	-3.9
	DP98140		0.6		0.4	

[a] conversion factor of 0.5 from EURL Technical guidance on implementation of EU Regulation 619/2011[1] was used to convert ddPCR results from copy number ratio to mass/mass ratio

[b] conversion factors determined based on experimental results (certified reference materials were subjected to absolute quantification with ddPCR and then copy number result was compared to the certified mass/mass %; for factors see Table S6) were used to convert ddPCR results from copy number ratio to mass/mass ratio

Table S10. List of all CRM test materials used in this study

Event	UI	Producer
DP98140	DP-Ø9814Ø-6	IRMM
GA21	MON-ØØØ21-9	IRMM
MIR162	SYN-IR162-4	AOCS
MIR604	SYN-IR6Ø4-5	IRMM
MON810	MON-ØØ810-6	IRMM
MON863	MON-ØØ863-5	IRMM
MON89034	MON-89Ø34-3	AOCS

UI: OECD unique identifier of the transgenic maize event.

IRMM: Institute for Reference Materials and Measurements, Geel, Belgium.

AOCS: American oil chemists' society.

Table S11. Methods used in this study

Event/construct/gene	UI	Multiplex group	Method reference	Amplicon size [bp]
hmgA	-	MTQ1	QT-TAX-ZM-002	79
DP98140	DP-Ø9814Ø-6	MTQ1	QT-EVE-ZM-021	102
MON810	MON-ØØ810-6	MTQ1	QT-EVE-ZM-020	92
MON863	MON-ØØ863-5	MTQ1	QT-EVE-ZM-009	84
GA21	MON-ØØØ21-9	MTQ2	QT-EVE-ZM-014	101
MIR162	SYN-IR162-4	MTQ2	QT-EVE-ZM-022	92
MIR604	SYN-IR6Ø4-5	MTQ2	QT-EVE-ZM-013	76
MON89034	MON-89Ø34-3	MTQ2	QT-EVE-ZM-018	77

UI: OECD unique identifier of the transgenic maize event.

Multiplex group: multiplex assay in which the event/gene is present.

Method reference: reference for the method for quantification of event. All methods were validated by the EURL-GMFF (http://gmo-crl.jrc.ec.europa.eu/gmomethods/).

Table S12. Specifications of stock DNA mix and all of the dilution series samples

		Copy numbers per reaction[a]								GM%[b]						
Sample	Dilution[c]	hmgA	DP98140	MON810	MON863	GA21	MIR162	MIR604	MON89034	DP98140	MON810	MON863	GA21	MIR162	MIR604	MON89034
DNA mix 1	Stock DNA	67575.6	1236.9	1183.8	936.5	766.9	887.4	802.8	836.5	1.8	1.8	1.4	1.1	1.3	1.2	1.2
DNA mix 2	3 x	22525.2	412.3	394.6	312.2	255.6	295.8	267.6	278.8	1.8	1.8	1.4	1.1	1.3	1.2	1.2
DNA mix 3	9 x	7508.4	137.4	131.5	104.1	85.2	98.6	89.2	92.9	1.8	1.8	1.4	1.1	1.3	1.2	1.2
DNA mix 4	27 x	2502.8	45.8	43.8	34.7	28.4	32.9	29.7	31.0	1.8	1.8	1.4	1.1	1.3	1.2	1.2
DNA mix 4a	40.5x	1668.5	30.5	29.2	23.1	18.9	21.9	19.8	20.7	1.8	1.8	1.4	1.1	1.3	1.2	1.2
DNA mix 4b	54x	1251.4	22.9	21.9	17.3	14.2	16.4	14.9	15.5	1.8	1.8	1.4	1.1	1.3	1.2	1.2
DNA mix 5	81 x	834.3	15.3	14.6	11.6	9.5	11.0	9.9	10.3	1.8	1.7	1.4	1.1	1.3	1.2	1.2
DNA mix 6	243 x	278.1	5.1	4.9	3.9	3.2	3.7	3.3	3.4	1.8	1.8	1.4	1.2	1.3	1.2	1.2
DNA mix 7	729 x	92.7	1.7	1.6	1.3	1.1	1.2	1.1	1.1	1.8	1.7	1.4	1.2	1.3	1.2	1.2
DNA mix 8	2187 x	30.9	0.6	0.5	0.4	0.4	0.4	0.4	0.4	1.9	1.6	1.3	1.3	1.3	1.3	1.3
DNA mix 9	6561 x	10.3	0.2	0.2	0.1	0.1	0.1	0.1	0.1	1.9	1.9	1.0	1.0	1.0	1.0	1.0
DNA mix 10	19683 x	3.4	0	0	0	0	0	0	0	0	0	0	0	0	0	0

[a] copy numbers in stock DNA were measured with simplex ddPCR. All other values (for dilutions 3 x – 19683 x) were calculated from stock value using the dilution factor. All these values are considered as assigned values, when calculating correlation between measured copy numbers per reaction with the two multiplex assays and assigned copy numbers per reaction on a DNA mixture of 7 maize events (as presented on Figure 1)

[b] GM% calculated as ratio of copy numbers for each of the events against copy numbers of hmgA endogene.

[c] 3-fold dilutions were used to prepare the dilution series

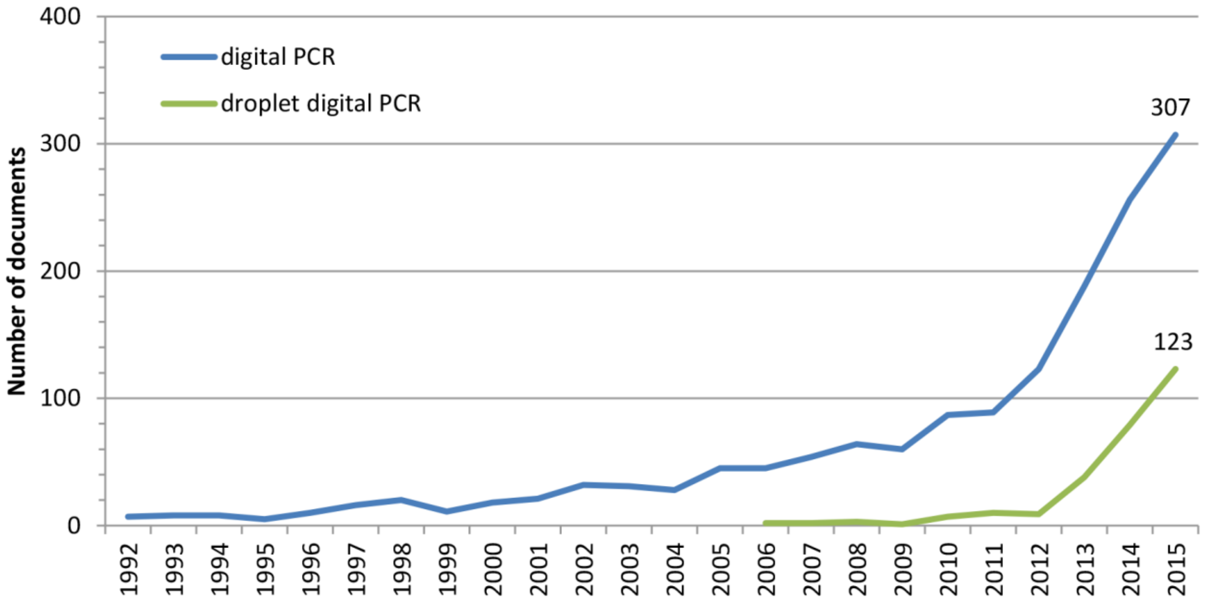

Figure S1. Number of publications containing “digital PCR” (blue line) or “droplet digital PCR” (green line) in title, abstract or key-words from 1992 to the end of 2015 gathered from “Analyze search results” module in Scopus database.

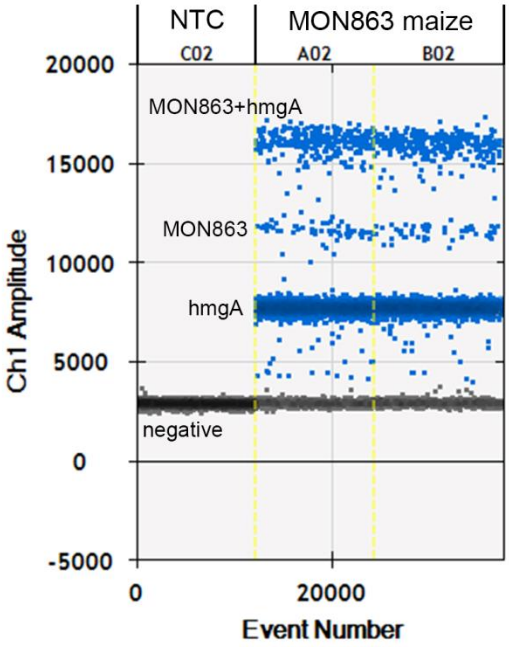

Figure S2. Example of duplex droplet readout in one fluorescence channel. hmgA had low probe concentration and MON863 had high probe concentration. The readout for three wells is shown: no template control (NTC) in well C02 and MON863 maize DNA in wells A02 and B02.

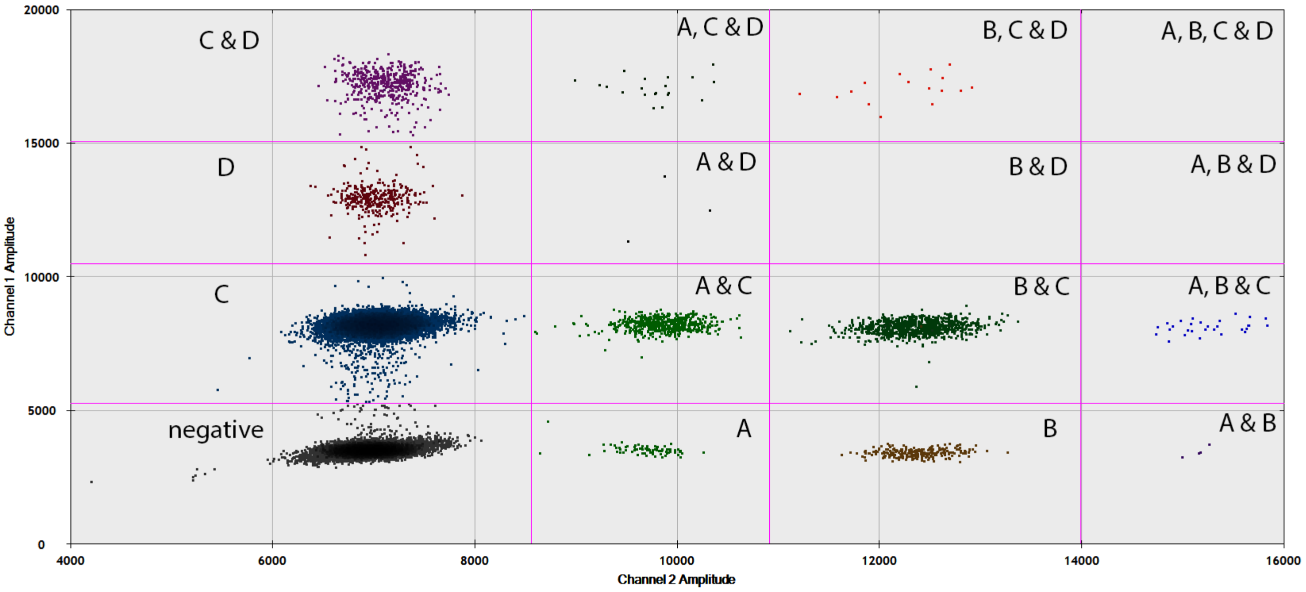

Figure S3. Graphical representation of possible target combinations in droplet clusters and their area of appearance in the 2-D amplitude view.

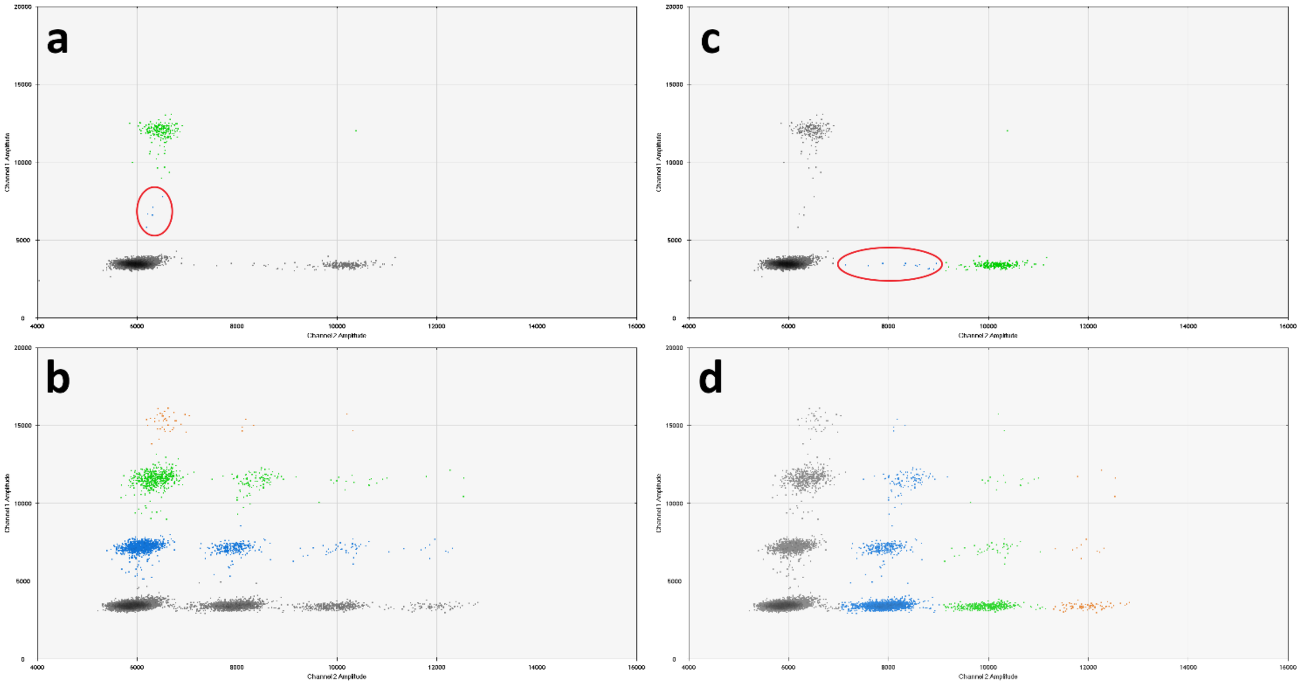

Figure S4. Example of false positive signal in the case of MTQ2 assay. GA21 and MIR604 DNA mixture was used (a and c) and mixture of DNA of 7 GM maize lines was used as positive control sample (b and d). In panels A (for GA21) and C for MIR604) clusters of positive droplets for specific targets are colored in green. Droplets colored in blue and marked with red ellipse are the false positive droplets (rain effect) as they fall into the space, where positive droplets of two other amplicons are expected, which can be determined based on the positive control output (B and D).

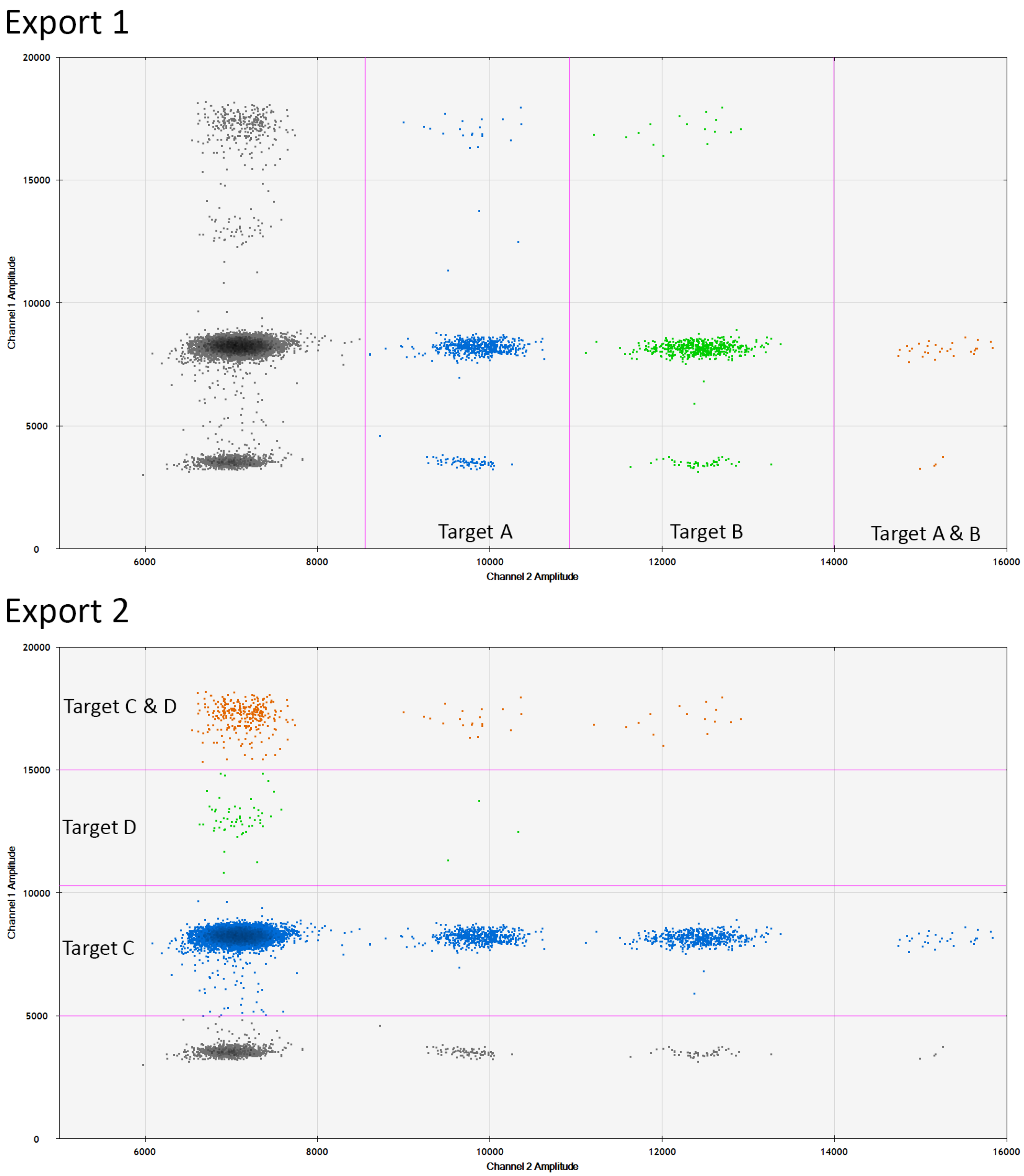

Figure S5. Example of cluster selection with the lasso tool. For each data export four clusters were selected with the lasso tool. In both exports (one for each fluorescent channel) the grey colored cluster represents negative droplets, blue colored cluster represents droplets positive for one target, green colored cluster represents droplets positive for second target and the orange colored cluster represents droplets positive for both targets.

After readout with ddPCR Droplet Reader, within the QuantaSoft software, select all the wells, for which you want to export the raw data. Under “Setup” tab, select “Options” and click on “Export Amplitude and Cluster Data”. Then select the folder on your computer in which the file will be exported. The files are exported in .csv file format.

The ddPCR multiplex web based tool can be accessed at http://bit.ly/ddPCRmulti.

This is the opening page of the ddPCR multiplex calculator:

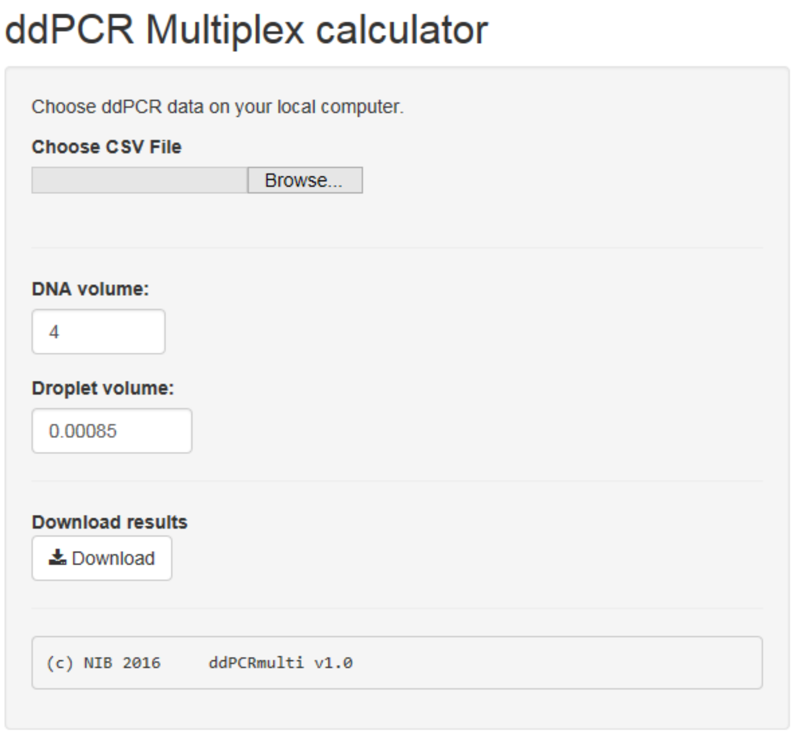

In the window “DNA volume” you have to insert the volume (in µl) of the DNA sample that was added to the reaction mixture (the total reaction volume is 20 µl).

In the window “Droplet volume” you can insert the actual droplet volume (in µl) in case you measured the actual volume of your droplets, otherwise you can use the default droplet volume of 0.00085 µl.

To start the analysis, you have to Choose the .csv file you want to analyze, by clicking on the “Browse…” button. After loading, the analysis will automatically be performed.

The tool will set the thresholds automatically between the clusters and count the number of positive and negative droplets for each of the targets (A, B, C, D).

Note that the automated threshold setting may be affected by shifted cluster position (example further down), by uneven droplet distribution within the clusters, or by the cluster shape itself. Ideally, all the thresholds should be set in the middle between the clusters like here (the ddPCR calculator is programmed to find this middle spot between the clusters):

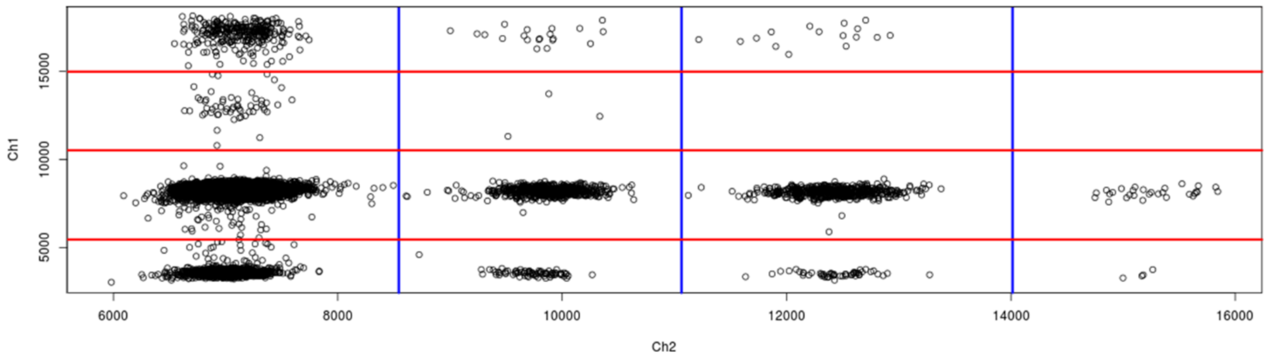

It is advisable to first perform the analysis of the positive control, where all, or at least the majority (like on the figure above), of the clusters should be present. With this analysis one can get the approximate threshold values that can be used as indicator thresholds in the analysis of the samples.

In case a threshold is not set exactly in the middle between clusters and there is a chance that few droplets get misclassified (usually in the cases of highly concentrated samples) to another cluster, this would not have a significant effect on the quantification results, since the number of other “real” positive droplets is so high that few misclassified droplets do not have any significant effect.

It is worthwhile to mention that between different batches of probes the measured fluorescence amplitude may be different. To illustrate, we have two such batches of MTQ1 assay, where the Ch1 thresholds were approximately at 5500, 10500 and 15000 for first one and 4000, 8000 and 10000 for the second batch. The same was observed with the Ch2 thresholds, where for the first batch they were at 8500, 11000 and 14000 and for the second batch at 7400, 9200 and 11300. This difference in thresholds, however, does not have an effect on the final quantification result, since the number of positive droplets is not affected, only their fluorescence amplitude is different. Additionally, the Droplet Reader calibration status may affect the amplitude fluorescence reported and measured by QuantaSoft software (in our lab a new calibration caused a shift in fluorescence for 200 units).

Whenever the threshold position after the automated analysis is not ok (see example below), you can modify the position of individual thresholds.

To modify the position of vertical thresholds, you have to click inside the plot close to the x-axis (see cross circled with green in upper panel of the example figure below, with the area highlighted in yellow) and the closest threshold line will be moved to this new position. To modify the position of horizontal thresholds, you have to click inside the plot close to the y-axis (see cross circled with green in upper panel of the example figure below with the area highlighted in yellow) and the closest threshold line will be moved to this new position. Whenever the tool detects change in threshold setting (or change in DNA or Droplet volume) the calculations are performed automatically using the new input values.

Below the plot there is a table with all the calculated values for each of the targets. The calculations include for each target the number of positive droplets, the number of total droplets, concentration (in copies per 20 µl reaction), lambda value, lambda values with 95% confidence intervals, input concentration (in copies per µl of sample DNA per effective reaction volume) and 95% confidence intervals for input concentration.

To export the table with results in a tab delimited .txt file, click on the “Download” button.

ddPCR Multiplex calculator

Choose ddPCR data on your local computer.

Choose CSV File

C:\Users\davidd\Desktop\ Browse...

Upload complete

DNA volume:

4

Droplet volume:

0.00085

Download results

Download

(c) NIB 2016 ddPCRmulti v1.0

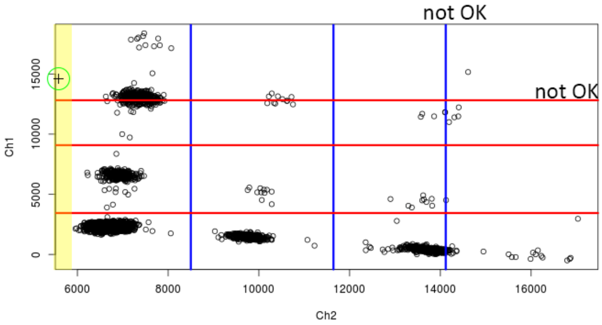

File: MEQ_E07_Amplitude.csv

	Positive	Total	Concentration	lambda	lambdaL	lambdaH	InputConc	InputConcL	InputConcH
A	447	12728	455	0	0	0	210	191	230
B	394	12728	400	0	0	0	185	167	203
C	734	12728	756	0	0	0	349	324	375
D	417	12728	424	0	0	0	196	177	215

ddPCR Multiplex calculator

Choose ddPCR data on your local computer.

Choose CSV File

C:\Users\davidd\Desktop\ Browse...

Upload complete

DNA volume:

4

Droplet volume:

0.00085

Download results

Download

(c) NIB 2016 ddPCRmulti v1.0

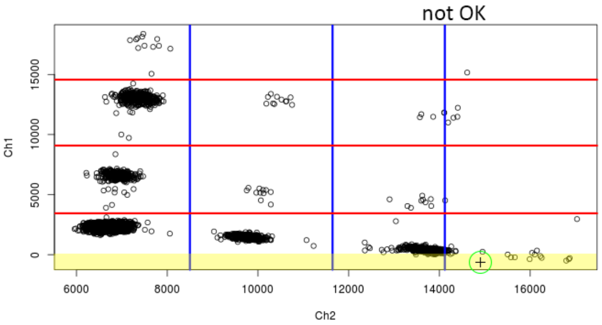

File: MEQ_E07_Amplitude.csv

	Positive	Total	Concentration	lambda	lambdaL	lambdaH	InputConc	InputConcL	InputConcH
A	447	12728	455	0	0	0	210	191	230
B	394	12728	400	0	0	0	185	167	203
C	447	12728	455	0	0	0	210	191	230
D	417	12728	424	0	0	0	196	177	215

ddPCR Multiplex calculator

Choose ddPCR data on your local computer.

Choose CSV File

C:\Users\davidd\Desktop\ Browse...

Upload complete

DNA volume:

4

Droplet volume:

0.00085

Download results

Download

(c) NIB 2016 ddPCRmulti v1.0

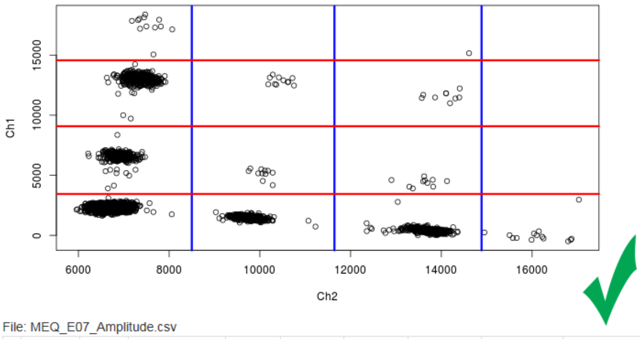

File: MEQ_E07_Amplitude.csv

	Positive	Total	Concentration	lambda	lambdaL	lambdaH	InputConc	InputConcL	InputConcH
A	425	12728	432	0	0	0	200	181	219
B	394	12728	400	0	0	0	185	167	203
C	447	12728	455	0	0	0	210	191	230
D	417	12728	424	0	0	0	196	177	215

Reference list

1. EURL-GMFF. Technical guidance document from the European Union Reference Laboratory for Genetically Modified Food and Feed on the implementation of Commission Regulation (EU) No 619/2011. http://gmo-crl.jrc.ec.europa.eu/doc/Technical Guidance from EURL on LLP.pdf (2011)
